# The Murine Coronavirus Hemagglutinin-esterase Receptor-binding Site: A Major Shift in Ligand Specificity through Modest Changes in Architecture

**DOI:** 10.1371/journal.ppat.1002492

**Published:** 2012-01-26

**Authors:** Martijn A. Langereis, Qinghong Zeng, Balthasar Heesters, Eric G. Huizinga, Raoul J. de Groot

**Affiliations:** 1 Virology Division, Department of Infectious Diseases and Immunology, Faculty of Veterinary Medicine, Utrecht University, Utrecht, The Netherlands; 2 Crystal and Structural Chemistry, Bijvoet Center for Biomolecular Research, Faculty of Sciences, Utrecht University, Utrecht, The Netherlands; Erasmus Medical Center, The Netherlands

## Abstract

The hemagglutinin-esterases (HEs), envelope glycoproteins of corona-, toro- and orthomyxoviruses, mediate reversible virion attachment to *O*-acetylated sialic acids (*O*-Ac-Sias). They do so through concerted action of distinct receptor-binding (“lectin”) and receptor-destroying sialate *O*-acetylesterase (”esterase”) domains. Most HEs target 9-*O*-acetylated Sias. In one lineage of murine coronaviruses, however, HE esterase substrate and lectin ligand specificity changed dramatically as these viruses evolved to use 4-*O*-acetylated Sias instead. Here we present the crystal structure of the lectin domain of mouse hepatitis virus (MHV) strain *S* HE, resolved both in its native state and in complex with a receptor analogue. The data show that the shift from 9-*O*- to 4-*O*-Ac-Sia receptor usage primarily entailed a change in ligand binding topology and, surprisingly, only modest changes in receptor-binding site architecture. Our findings illustrate the ease with which viruses can change receptor-binding specificity with potential consequences for host-, organ and/or cell tropism, and for pathogenesis.

## Author Summary

Glycans cover the surface of every living cell. In vertebrates, these sugar trees commonly terminate with sialic acid (Sia) and, in consequence, Sias have become the attachment factors of choice for a multitude of pathogens: protozoa, bacteria and viruses alike. To ensure selectivity, viruses evolved to target distinct Sia species. Whether a particular type of Sia serves as receptor may depend -amongst others- on the absence or presence of specific Sia modifications. For example, most group A betacoronaviruses attach to 9-*O*-acetylated Sias. However, some murine coronaviruses have switched to using 4-*O*-acetylated Sias instead. In chemical/molecular terms this represents a momentous shift in receptor usage. We now have crystallized the hemagglutinin-esterase protein (HE) of a murine coronavirus and have solved the structure of its sugar-binding domain. Our findings reveal in exquisite detail the interactions between Sia binding site and cognate receptor. The data allow a reconstruction of how, during coronavirus evolution, the switch in receptor usage may have come about.

## Introduction

To initiate infection viruses must bind to an appropriate host cell. Selectivity of binding is ensured by attachment proteins on the virion, tailored to recognize one -or at the most- a limited number of cell surface molecules. Remarkably, a large number of viruses, representative of at least 11 distinct families several of which of clinical and/or veterinary importance, use sialic acid (Sia) as receptor determinant. Owing to differential modification, Sia structural diversity exceeds that of any other monosaccharide [1]. The most common type of Sia substitution, *O*-acetylation at carbon atoms C4, C7, C8 and/or C9, occurs in a host-, organ- and even cell-specific fashion such that even individual cells of the same type and tissue may differ in their Sia expression profile [2]–[4]. Viruses have evolved to selectively use particular Sia variants and their attachment proteins are high-specificity sialolectins, the binding of which might depend on the identity of the penultimate residue in the sugar chain, the type of glycosidic linkage and/or the presence or absence of substitutions [5]–[9]. Ultimately, this preference in Sia receptor usage affects host-, organ-, and cell-tropism [10]–[14], the course and outcome of infection [15]–[18] as well as the efficacy of intra- and cross-species transmission [14], [19], all to extents not yet fully appreciated.

**Figure 1:**
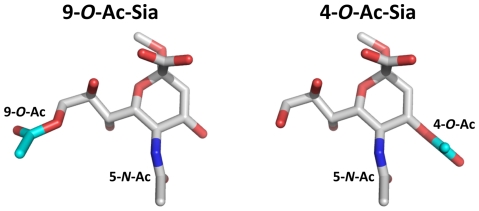
Stereochemical differences between 9-*O*- and 4-*O*-acetylated sialic acid. Stick representation of (*left*) αNeu5,9Ac_2_2Me and (right) αNeu4,5Ac_2_2Me. Backbone αNeu5Ac2Me is colored in gray (carbon), red (oxygen) and blue (nitrogen). The 9-*O*-Ac group of αNeu5,9Ac_2_2Me and 4-*O*-Ac group of αNeu4,5Ac_2_2Me are highlighted in cyan (carbon).

The hemagglutinin-esterases (HEs) are a class of Sia-binding envelope glycoproteins found in some negative-stranded RNA viruses, namely in influenza C and infectious salmon anemia virus (family *Orthomyxoviridae*; [5], [20]), but also in toro- and coronaviruses, positive-stranded RNA viruses in the order *Nidovirales* [21], [22]. From phylogenetic and comparative structural analyses it appears that toro- and coronaviruses acquired their HE proteins separately via horizontal gene transfer, with an (hemagglutinin-esterase-fusion) HEF-like protein as progenitor [22]–[25]. Like influenza C virus HEF, most nidovirus HEs bind to 9-*O*-acetylated (9-*O*-Ac) Sias and, correspondingly, display sialate-9-*O*-acetylesterase receptor-destroying enzyme activity [25]. Murine coronaviruses, however, occur in two closely related biotypes that differ in HE ligand/substrate preference. One of these -represented by mouse hepatitis virus (MHV) strain DVIM- displays the presumptive ancestral specificity and targets 9-*O*-Ac-Sias, while the other -represented by MHV strain *S*- appears to have evolved to use 4-*O*-Ac-Sias instead [6], [25]–[27] (for supplementary introduction see Text S1 and Figure S1). Given the stereochemical differences between these Sia variants (Figure 1) and the essentially different requirements for ligand and substrate recognition by the respective HEs, the question arises how this major shift in receptor usage was achieved and what changes must have occurred in the receptor-binding and *O*-acetylesterase domains to make this transition possible.

The crystal structures of a number of 9-*O*-Ac-Sia-specific nidovirus HEs have been solved [23], [24]. Unlike the receptor-binding site (RBS) of influenza C virus HEF [28], the RBSs of the corona- and torovirus HEs seem to be exceptionally plastic as they appear to have undergone significant changes and adaptations that altered their overall architecture in a relatively short evolutionary time span. Based on these observations, we anticipated and speculated [23] that this plasticity might have allowed for even more substantial adjustments in the RBS of the murine coronavirus HE as to produce an entirely novel binding site specific for 4-*O*-acetylated Sias.

We now present the crystal structure of the MHV*-S* HE receptor-binding domain, both in its native state and in complex with a receptor analogue. The data reveal in exquisite detail how the RBS changed to accommodate 4-*O*- instead of 9-*O*-acetylated Sias. Surprisingly, however, this shift in receptor usage seems to have involved primarily a change in ligand binding topology and relatively modest changes in RBS architecture.

## Results/Discussion

### Expression, purification, and biochemical characterization of MHV*-S* HE

**Figure 2:**
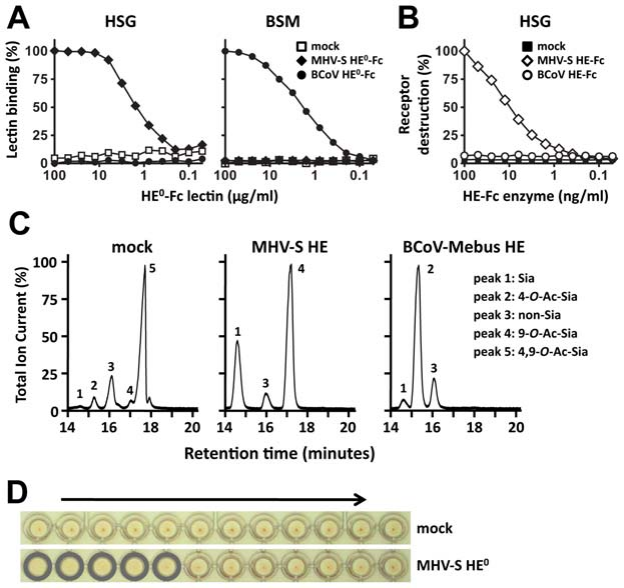
HE-Fc fusion protein displays proper receptor-binding and enzymatic activities. (A) Binding of two-fold serial dilutions (starting at 100 µg/ml) of esterase-deficient Fc-fusion proteins (HE^0^-Fc) of BCoV-Mebus and MHV*-S* in a solid-phase lectin-binding assay towards horse serum glycoproteins (HSG) and bovine submaxillary mucins (BSM). Relative binding in percentages is calculated with the binding of the highest concentration lectin set at a 100%. Wells incubated without lectin (“mock”) were included as negative control. (B) Receptor destroying enzyme activity towards HSG. Coated HSG was treated with two-fold serial dilutions (starting at 100 ng/ml) of enzymatically-active BCoV-Mebus and MHV-*S* HE Fc-fusion proteins and 4-*O*-Ac-Sia content was detected by solid phase lectin binding assay with MHV-*S* HE^0^-Fc. Decrease in signal as compared to untreated HSG is plotted in percentages. (C) MHV-*S* HE ectodomain displays sialate-4-*O*-acetylesterase activity towards the synthetic di-*O*-acetylated sialic acid analogue αNeu4,5,9Ac_3_2Me. Graphs show total ion current gas-chromatograms and Sia subtypes were identified by mass spectrometry: Sia (αNeu5Ac2Me [peak 1]), 4-*O*-Ac-Sia (αNeu4,5Ac_2_2Me [peak 2]), 9-*O*-Ac-Sia (αNeu5,9Ac_2_2Me [peak 4], 4,9-di-*O*-Ac-Sia (αNeu4,5,9Ac_3_2Me [peak 5]). Peak 3 represents a non-sialic acid compound. (D) Receptor binding activity of MHV-*S* HE ectodomain was assessed by hemagglutination assay with rat erythrocytes and twofold serial dilutions of the HE proteins (10,000 to 5 ng per well, arrow).

We produced the ectodomain of MHV*-S* HE (residues 25-403) as an Fc-fusion protein, either in enzymatically active (HE-Fc) or inactive form (HE^0^-Fc), by transient transfection of HEK293 cells. MHV*-S* HE^0^-Fc bound to horse serum glycoproteins (HSG), which are decorated with 4-*O*-acetylated sialic acids (4-*O*-Ac-Sia), but carry little to no 9-*O*-Ac-Sias (Figure 2A; [29]). The receptor determinants in HSG could be destroyed by treatment with MHV*-S* HE-Fc, but not by treatment with BCoV-Mebus HE-Fc (a sialate-9-*O*-acetylesterase; Figure 2B). No binding of MHV*-S* HE^0^-Fc was observed to bovine submaxillary mucin (BSM), a glycoconjugate devoid of 4-*O*-Ac-Sias (Figure 2A; [30]). The MHV*-S* HE ectodomain, released from HE-Fc by thrombin-cleavage, retained proper sialate-4-*O*-acetylesterase activity when assayed for substrate specificity with a synthetic di-*O*-acetylated Sia (5-*N*-acetyl-4,9-di-*O*-acetylneuraminic acid α-methylglycoside, αNeu4,5,9Ac_3_2Me; Figure 2C). In hemagglutination assays, MHV*-S* HE^0^ specifically bound to 4-*O*-acetylated Sias (Figure 2D). The combined findings show that the recombinant MHV*-S* HE proteins are biologically active, both as Fc fusion proteins (Figures 2A and B) and after the removal of the Fc tail by thrombin-cleavage (Figures 2C and D), which we take as an indication for proper folding and protein stability.

### Structure determination and overall structure

Crystals of free MHV*-S* HE and of a complex of HE^0^ with αNeu4,5Ac_2_2Me diffracted to 2.1 and 2.5 Å resolution, respectively. The structures were solved by molecular replacement by using BCoV-Mebus HE (PDB ID 3CL5; [23]) as template (BCoV-Mebus and MHV*-S* HE share 59% sequence identity; for crystallographic details, see Table 1).

**Table 1:** Data collection and refinement statistics.

	MHV-S HE	MHV-S HE^0^ with ligand
**Data collection**		
Spacegroup	*P2_1_2_1_2_1_*	*P2_1_2_1_2_1_*
Cell dimensions *a,b,c* (Å)	92.8,108.8,125.1	91.6,106.6,135.6
α,β,γ (^o^)	90.0, 90.0, 90.0	90.0, 90.0, 90.0
Resolution (Å)*	30-2.1	54.5-2.5
	(2.22-2.10)	(2.64-2.50)
Completeness (%)	99.5 (96.8)	100.0 (100.0)
#Unique reflections	73858(10352)	46670(6706)
Multiplicity	7.4(7.4)	7.4 (7.4)
R_merge_ (%)	10.3 (70.1)	12.2 (90.2)
I/σ	12.9 (2.9)	12.7 (2.1)
**Refinement**		
R_work_/R_free_ † (%)	18.8/22.2	21.3/24.9
#Protein atoms‡	5676	5457
#Glycan units	31	28
#Waters	291	77
Mean B value protein (Å^2^)	33.4	28.8
Mean B value water (Å^2^)	38.5	28.4
Rmsd bond lengths (Å)	0.010	0.009
Rmsd bond angles (°)	1.3	1.2
**Ramachandran plot**		
Favored regions (%)	94.6	95.0
Allowed regions (%)	4.7	4.4
Disallowed regions (%)	0.7	0.6

*Values between brackets refer to the highest resolution shell of data.

**†:** Rfree is calculated from 5% of data randomly chosen not to be included in refinement.

**‡:** Two HE molecules are present in the asymmetric unit of the crystal; during refinement no NCS restraints were applied.

**Figure 3:**
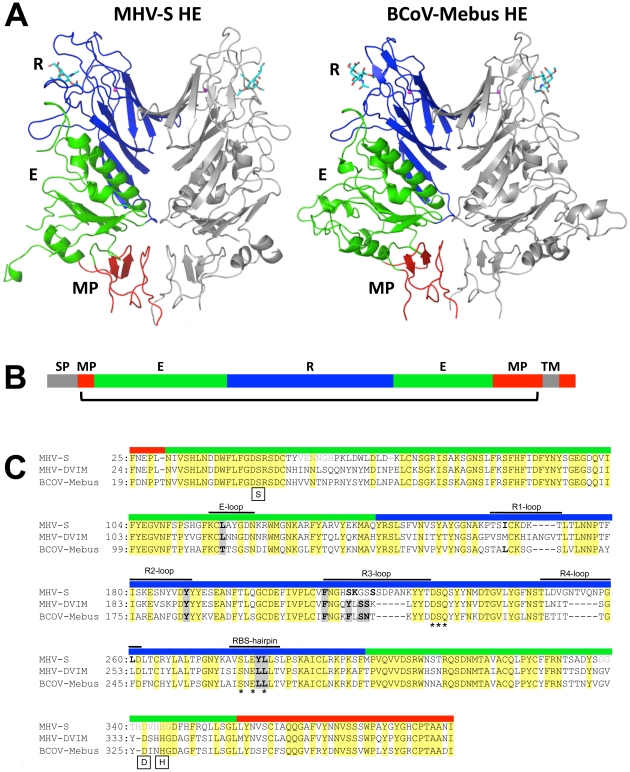
Overall structure and comparison to BCoV-Mebus HE. (A) Ribbon representation of the dimeric MHV*-S* (residues 25–395) and BCoV-Mebus HE (residues 19–376) structures. One monomer is colored grey, the other by domain: lectin domain (R, blue) with bound αNeu4,5Ac_2_2Me (MHV*-S* HE) or αNeu4,5,9Ac_3_2Me (BCoV-Mebus HE; cyan sticks) and potassium ion (magenta sphere); esterase domain (E, green); membrane-proximal domain (MP, red). (B) Linear representation of MHV HE with domains color-coded as in panel A. Grey segments indicate the signal-peptide (SP) and transmembrane (TM) domain. The bracket indicates the part of the protein for which the structure has been solved. (C) Structure- (MHV*-S* and BCoV-Mebus) and sequence-based (MHV-DVIM) alignment of HE sequences. Colored boxes above the sequences indicate domain organization as in panel A and B and black lines indicate loops involved in receptor binding. Note that in MHV-*S* HE two insertions increase the length of loops R3 and R4. Asterisks indicate the highly conserved residues of the potassium binding site and boxes indicate the critical serine, histidine and aspartic acid residues of the catalytic site. Residues that interact with the ligand are indicated in bold; those conserved among all three HEs are highlighted by grey shading. Other residues also conserved in all three HEs are highlighted in yellow. The residues in disordered loops of the esterase domain are indicated in light gray lettering.

In overall structure, the HE of MHV*-S* closely resembles that of BCoV-Mebus. It assembles into homodimers and the monomers are composed of three modules: a small membrane-proximal (MP), a receptor-binding (R), and a central esterase (E) domain (Figures 3A–C; [23]). The MP domain is virtually identical to that of BCoV-Mebus HE with a root mean square difference (rmsd) on main chain Cα atoms of only 0.48 Å. Unfortunately, residues in the E domain, that form the catalytic site were disordered in both crystals. Hence, the molecular basis for the unusual substrate specificity of MHV*-S* HE remains unknown. The structure of the R domain, however, was resolved, and in the complex the ligand molecule is well-defined (Figure S2). The R domains of MHV*-S* and BCoV-Mebus HE are highly similar with an rmsd on main chain Cα atoms of 0.79 Å.

### MHV*-S* HE has a unique receptor binding-site

**Figure 4:**
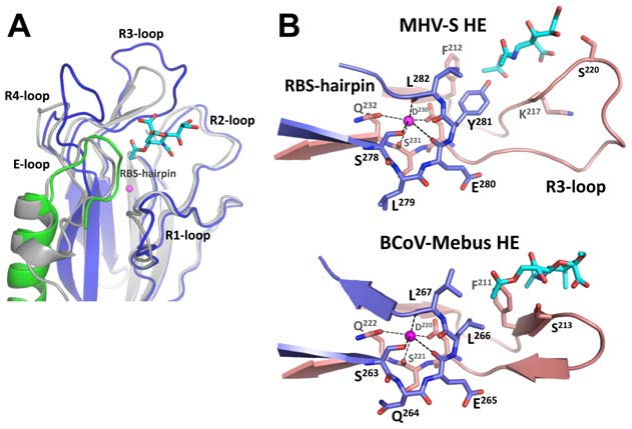
Comparison of the MHV-S and BCoV-Mebus HE receptor binding sites. (A) Ribbon superposition of the MHV-*S* and BCoV-Mebus HE receptor binding sites. BCoV-Mebus HE is colored gray, coloring of MHV-*S* HE as in panel A. Bound receptor analogues are shown as cyan sticks and potassium ions as magenta spheres. The five surface exposed loops and the RBS-hairpin that interact with the receptor are indicated. Note that only the R3- and R4-loops differ in conformation. (B) Close-up of the HE-potassium binding-site of MHV*-S* HE and BCoV-Mebus HE. Shown in ribbon representation are the R3-loop (salmon) and RBS-hairpin (purple) that interacts with the potassium ion (magenta sphere).

The receptor-binding sites of BCoV-Mebus and MHV*-S* HE are very much alike in architecture. This is particularly surprising given the considerable differences in ligand preference and in their requirements for binding (i.e. binding of 9-*O*-Ac-Sia in a 9-*O*-Ac-dependent fashion versus binding of 4-*O*-Ac-Sia in 4-*O*-Ac-dependent fashion, respectively; Figure 1). The MHV*-S* HE receptor-binding site (RBS), like that of BCoV-Mebus HE, is composed of 5 surface exposed loops, four of which extend from the conserved 8-stranded “Swiss role” core-structure (loops R1 through R4; Figures 3C and 4A) and one originating from the E-domain (E-loop). Whereas the R1-, R2- and E-loops of the BCoV-Mebus and MHV*-S* HE sites are almost identical, the R3- and R4-loops adopt different conformations in the two proteins as result of amino acid insertions in MHV HE (Figures 3C and 4A). Two other conspicuous elements of the MHV*-S* HE RBS are the RBS-hairpin and a conserved metal-binding site with a potassium ion that stabilizes the R3-loop and the RBS-hairpin exactly as in BCoV-Mebus HE (Figure 4B). The potassium ion is coordinated by main chain oxygen atoms of Ser^231^, Glu^280^ and Leu^282^ and side chain oxygen atoms of Asp^230^, Gln^232^ and Ser^278^. These residues are conserved in BCoV-Mebus HE and in all other coronavirus HEs with the exception of HCoV-HKU1 HE [23].

**Figure 5:**
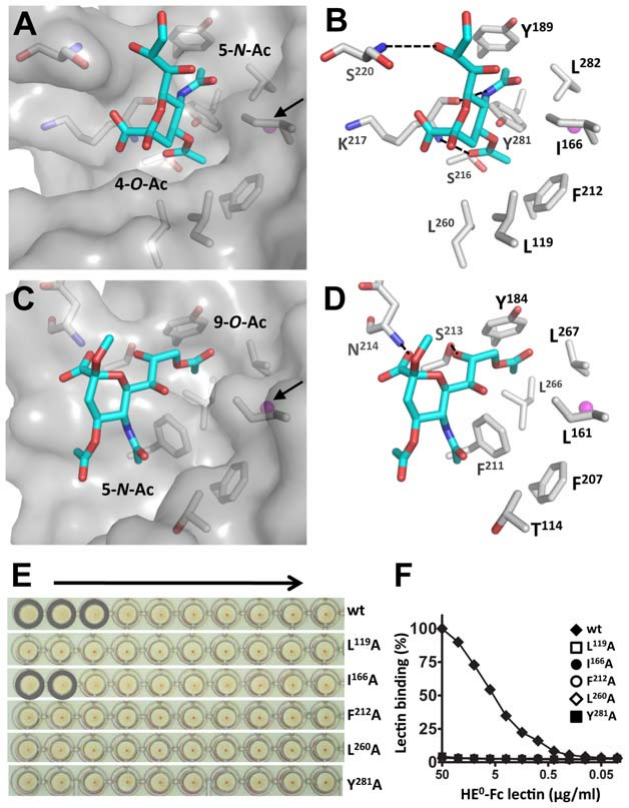
MHV*-S* HE has a unique receptor-binding site that binds specifically 4-*O*-acetylated sialic acid. (A) Surface and (B) stick representation of the MHV*-S* HE receptor-binding site in complex with a receptor analogue. The ligand bound to the HE receptor-binding site is shown in stick representation and the potassium ion as a magenta sphere, indicated by a black arrow in panel A. Hydrogen bonds between HE and the receptor are shown as black dashed lines. Surface representation of the MHV*-S* HE receptor-binding site reveals two pockets accommodating the 4-*O*- and 5-*N*-acetyl groups of the receptor, respectively. Note that crystals were soaked with αNeu4,5,9Ac_3_2Me, but most likely as a result of the low pH crystallization conditions, the 9-*O*-Ac group was lost [42]. (C) Surface and (D) stick representation of the BCoV-Mebus HE receptor-binding site. Note that the topology of the two hydrophobic pockets is conserved, except they bind different substituents of the receptor analogue. (E) The effect of Ala substitutions on receptor binding. Relative binding affinity of wild-type HE^0^ (wt) and its derivatives was assessed by hemagglutination assay with rat erythrocytes and twofold serial dilutions of each of the HE^0^-Fc chimeras (5,000 to 10 ng per well, arrow). (F) Binding of twofold serial dilutions of wild-type (wt) HE^0^-Fc chimera and its derivatives in a solid-phase lectin-binding assay towards horse serum glycoproteins (HSG) as described in Figure 2A.

While the overall organization of the MHV*-S* RBS is similar to that of BCoV-Mebus HE, the orientation of the receptor analogue with respect to the RBS is strikingly different (Figures 4B, 5A and B). As compared to the ligand in the BCoV-Mebus HE binding site (Figures 5C and D), the αNeu4,5Ac_2_2Me receptor analogue is rotated by about 90° and shifted by about 2.5 Å. Figures 5A and B show how residues from the four R-loops, the E-loop and the RBS-hairpin interact with the Sia receptor molecule. Two hydrogen bonds are formed between the nitrogen and oxygen main-chain atoms of Lys^217^ and the oxygen of the C4 acetyl group and the nitrogen of the 5-*N*-acetyl group, respectively. The Ser^220^ main chain nitrogen accepts an additional, weak hydrogen bond from the C8 hydroxyl group of the ligand (Figure 5B).

Most remarkably, the hydrophobic pocket that in BCoV-Mebus HE accommodates the 9-*O*-acetyl moiety of the receptor (comprised of Leu^161^, Tyr^184^ Leu^266^ and Leu^267^) -arguably the most crucial element of the BCoV HE RBS- is conserved in MHV*-S* HE (comprised of Ile^166^, Tyr^189^, Tyr^281^, and Leu^282^), but it now accepts the Sia 5-*N*-acetyl group, while the Sia glycerol side-chain is solvent exposed (Figure 5A). Moreover, the hydrophobic patch in the BCoV-Mebus HE RBS that interacts with the Sia 5-*N*-acetyl group (Figure 5C) apparently changed into a shallow pocket that accommodates the Sia 4-*O*-acetyl moiety (Figure 5A). The residues orthologous to BCoV-Mebus HE Thr^114^, Leu^161^, Phe^211^, and Leu^266^ were replaced by Leu^119^, Ile^166^, Ser^216^, and Tyr^281^, respectively, and Leu^260^ was recruited from the R4-loop, which in MHV*-S* HE is reoriented as compared to the one in BCoV-Mebus HE (Figure 4A). These residues, together with conserved Phe^212^, form the hydrophobic lining of the newly shaped pocket (Figures 5A and B). As the Sia-4-*O*-acetyl group is crucial for ligand recognition by MHV*-S* HE, this pocket must be key to receptor-binding. In accordance, single Ala substitutions of Leu^119^, Ile^166^, Phe^212^, Leu^260^, or Tyr^281^ all reduced receptor-binding activity (although that of Ile^166^ to lesser extent) as shown by hemagglutination assay (Figure 5E) and solid-phase lectin binding assay (Figure 5F).

### Relatively modest changes in the MHV*-S* receptor binding-site changes ligand specificity

The data reveal in minute detail not only the mode of interaction between MHV*-S* HE and its cognate receptor determinant, but also clarify how a CoV HE RBS for 9-*O*-Ac-Sia might have transformed into one that now specifically binds 4-*O*-Ac-Sia. The most striking observation is that this major shift in ligand specificity required only minimal changes in the protein and that the binding site architecture was essentially maintained. How this was possible can be explained from the mode of lectin-ligand interaction, based largely on the docking of the methyl groups of the Sia-acetyl moieties into hydrophobic pockets, and from the structures of the two types of ligands. The juxtaposition of the Sia 5-*N*- and 9-*O*-acetyl moieties is quasi-similar to that of the Sia 4-*O*- and 5-*N*-acetyl groups. The distance between the groups may be different (7.1 versus 5.7 Å as measured between the methyl carbon atoms, respectively), but for each combination the acetyl groups are located in roughly the same plane and at roughly similar angles (Figure S3). Thus, it can be envisaged that a pre-existing site for 9-*O*-Ac-Sia was converted to accommodate 4-*O*-Ac-Sia instead by (i) having the ligand rotate (with binding of the ligand in the novel orientation facilitated through hydrogen bonding with residues introduced by substitutions and/or insertions in the R3 loop) and (ii) by bringing the original 9-*O*-acetyl binding pocket and 5-*N*-acetyl binding patch more closely together so that they now can accept the 5-*N*- and 4-*O*-acetyl moieties, respectively (Figure S3). From attempts to fit αNeu5,9Ac_2_2Me into the MHV*-S* RBS by *in silico* modelling, the the 9-*O*- and 5-*N*-acetyl groups would seem to be spaced too far apart to conveniently dock into the acetyl-binding pockets. Moreover, were the ligand to bind in this orientation, the Sia carboxylate would clash with the modified R3-loop. These findings thus provide an explanation for exclusion of the original ligand and for the specificity of MHV*-S* HE for 4-*O*-Ac-Sias (Figure S3 and Video S1).

The structure of the MHV*-S* HE-receptor complex allows guarded predictions only of how glycosidic linkage or additional Sia modifications might affect ligand binding. The C2-oxygen through which glycosidically-bound Sia would be linked to the penultimate residue of the glycan chain is exposed to the solvent and we would therefore expect the lectin to bind Sias in a linkage-independent fashion. Still, the R4- and/or E-loops, as they are proximal to Sia C2 (Figure 4A), might affect ligand binding such as to cause a preference for a particular linkage type. The pocket for the Sia 5-*N*-acetyl group would seem sufficiently wide to also accommodate the slightly larger 5-*N*-Gc substituent (Figure 5A); whether the lectin does accept 5-*N*-glycolylated Sias as ligands remains to be shown, however. Finally, from the topology and orientation of αNeu4,5Ac_2_2Me in the RBS of MHV*-S* HE, ligand binding would seem to be tolerant to modifications at the Sia glycerol side chain (Figure 5A). Yet, as demonstrated by hemagglutination assay with native and sialate-9-*O*-acetylesterase-treated erythrocytes, MHV*-S* HE apparently prefers 4-mono-*O*- over 4,9-di-*O*-acetylated Sias [27].

The occurrence of two distinct MHV lineages –exemplified by strains *S* and DVIM– that through their HE proteins bind to widely different Sia subtypes poses an interesting conundrum. While the structure reported here provides clues to how an HE protein ancestral to that of MHV*-S* may have changed to bind to 4-*O*- rather than to 9-*O*-acetylated Sias, the conditions that selected for this shift in ligand specificity and the biological consequences thereof are unknown. The limited data available on the *in vivo* role of HE suggests that it promotes viral spread [31]. Entry of murine coronaviruses, however, is mediated not by HE, but by the S protein, a type I fusion protein that binds to the principal receptor CAECAM1a [32]–[34]. We propose that HE may act during the very early stages of the infectious cycle as a molecular timer for temporary virion attachment. Through the concerted actions of its lectin and sialate-*O*-acetylesterase domains, HE would allow virus particles to bind with high avidity and yet reversibly to sialylated surfaces. The time allowed for virions to remain attached would be a function of HE binding affinity/avidity, esterase activity and local Sia density. Virions by binding to the ubiquitous and highly accessible Sias in the glycocalix would buy time for the S protein to find and bind the main receptor at the cell's surface as an obligatory prelude to penetration. Such a strategy would be advantageous particularly under conditions of low receptor density or poor receptor accessibility. If within the allotted time, HE-mediated virion attachment would not progress to this next stage of entry (for example, because the particle attached not to a susceptible cell, but to decoy receptors on a non-cell-associated glycoconjugate), the default would be for the virus to elute and “take its business elsewhere”. In this model, , MHV HE would appreciably contribute to host cell selection, its ligand preference potentially affecting host-, organ- and cell tropism. Our findings pave the way to study the function of CoV HE and to assess the importance of ligand and substrate specificity through an approach of structure-guided mutagenesis, reverse genetics and animal experimentation in a natural infection model.

## Materials and Methods

### Protein expression and purification

A synthetic DNA with human codon-optimized sequence for the HE ectodomain of MHV strain *S* (MHV*-S*; amino acid residues 25–403) was cloned in pCD5-Ig [23], [24], a derivative of expression plasmid S1-Ig [35]. The resulting construct, pCD5-MHV-*S*-HE-T-Fc, codes for a chimeric HE protein provided with an N-terminal CD5 signal peptide and, at its C-terminus, preceded by a thrombin cleavage site, the Fc domain of human IgG1 (HE-Fc). The QuikChange XL II site-directed mutagenesis kit (Stratagene) was used to construct pCD5-MHV-*S-*HE-T-Fc derivatives that code for an enzymatically inactive HE-Fc with the esterase catalytic residue Ser^45^ replaced by Ala (HE^0^-Fc), and for HE^0^-Fc mutants with Ala substitutions in the receptor-binding site. For analytical purposes, HE-Fc fusion proteins were produced by transient expression in HEK293T cells and then purified from the cell culture supernatants by protein A-affinity chromatography and low-pH elution (0.1M Citric-acid pH 3.0). The pH of the eluate was neutralized by adding Tris pH 8.0 to a final concentration of 0.2 M and the protein solution was dialyzed against phosphate-buffered saline (PBS). For crystallography, HE-Fc fusion-proteins were transiently expressed in HEK293 GnTI(-) cells [36] and the MHV*-S* ectodomain was purified by protein A-affinity chromatography and on-the-beads thrombin cleavage as described [23], [24].

### Solid-phase lectin binding assay (SLBA)

Maxisorp 96-well plates (NUNC) were coated for 16 hrs at 4°C with horse serum glycoproteins (HSG; 10% v/v horse serum in PBS) or bovine submaxilary mucin (BSM; 10 mg/ml; Sigma) at 100 µl per well. The wells were washed with washing buffer (PBS, 0.05% Tween-20) and treated with blocking buffer (PBS, 0.05% Tween-20, 2% bovine serum albumin, BSA) for 1 hr at RT. Two-fold serial dilutions of HE^0^-Fc lectins were prepared in blocking buffer (starting concentration 100 µg/ml) and 100 µl samples of these dilutions were added to the glycoconjugate-coated wells. Incubation was continued for 60 min after which unbound lectin was removed by washing three times. Bound lectin was detected using an HRP-conjugated goat anti-human IgG antiserum (1∶10,000 in blocking buffer; Southern Biotech) and TMB Super Slow One Component HRP Microwell Substrate (BioFX) according to the instructions. The staining reaction was terminated by addition of 0.3 M phosphoric acid, the optical density was measured at 450 nm, and graphs were constructed using GraphPad software. To assess and compare the enzymatic activities of BCoV-Mebus and MHV*-S* HE-Fc towards 4-*O*-acetylated Sias, HSG coated in Maxisorp plates was treated with samples from two-fold serial dilutions of either enzyme (starting at 100 ng/ml in PBS, 100 µl/well) for 2 hrs at 37°C. The destruction of 4-*O*-Ac-Sia receptor determinants was determined by SLBA with MHV*-S* HE^0^-Fc (5 µg/ml in blocking buffer) as described above. Enzymatic de-*O*-acetylation of αNeu4,5,9Ac_3_2Me was analyzed by gas-chromatography-electron impact mass-spectrometry (GC-MS) as described [24], [25], [37].

### Hemagglutination assay

Hemagglutination assay was performed in V-shaped 96-well plates (Greiner Bio-One). Two-fold serial dilutions in 50 µl PBS, 0.1% BSA of HE^0^-Fc or of purified HE^0^ ectodomains (starting amounts indicated in the text) were mixed with 50 µl of a rat erythrocyte suspension (*Rattus norvegicus* strain Wistar; 0.5% in PBS) and incubated for 2 hours on ice.

### Crystallization

Crystallization conditions were screened by the sitting-drop vapor diffusion method using a Honeybee 961 (Genomic Solutions). Drops were set up with 0.2 µl of HE protein solution in 10 mM Tris-HCl pH 8.0 and 0.2 µl reservoir solution. Crystals with space group *P2_1_2_1_2_1_* were obtained from 0.2 M KH_2_PO_4_, 0.2 M sodium malonate, 15% (w/v) PEG3350 and 0-5% (w/v) glycerol at 18°C. Crystals for diffraction experiments were grown with the hanging drop vapor diffusion method set up by hand with reservoir and protein solution ratio 1∶1 (1.6 µl total) at 18°C, and grew to a final size of up to 0.25×0.20×0.20 mm within one week. For data collection, crystals were flash-frozen in liquid nitrogen using reservoir solution containing 20% (w/v) glycerol as the cryoprotectant. To determine the HE structure in complex with its receptor, crystals of HE^0^ were soaked by adding 2 µl of 10 mM αNeu4,5,9Ac_3_2Me in cryoprotectant solution directly into the margin of the drop, resulting in a final substrate concentration of about 7 mM. Crystals were flash-frozen after 5 to 10 minutes.

### Data collection and structure solution

Diffraction data of crystals of MHV-S HE and its complex (Table 1) were collected at ESRF station ID-14-1 and ID-14-3, respectively. Diffraction data of native and ligand-soaked HE crystals were processed using XDS [38] and scaled using SCALA from the CCP4 suite [39]. Molecular replacement was performed using PHASER with BCoV-Mebus HE as template (PDB ID: 3CL5; [23]). Models were built manually with Coot [40] and refinement was carried out using REFMAC [41]. Water molecules were added using ARP/WARP, graphics generated with PYMOL (http://pymol.sourceforge.net).

In the Ramanchandran plot three residues are found in disallowed regions. The electron density of these residues supports the modeled conformation. In both HE monomers present in the asymmetric unit of the crystal structure of free as well as ligand-bound HE, the active site region of the esterase domain is largely disordered. No electron density is observed for esterase domain residues A52-A59, B51-B59, A108-A114, A308-A314, A335-A347 and B338-B346, while residues 44-50, 60-72, 332-334, and 348-358 adopt different conformations in the two monomers. Modeling of chain A residues 397-401 and chain B residues 334-337 and 394-398 should be considered tentative. C-terminal residues 396-403 followed by the 7-residue thrombin recognition sequence of the cleavable Fc-fusion are stabilized by crystal packing interactions suggesting that the observed conformation is not physiologically relevant.

## Supporting Information

Figure S1 **Rooted Neighbor-Joining tree depicting the evolutionary relationships among coronavirus HE proteins with Influenza C virus (IFC) HEF as outgroup.** Confidence values calculated by bootstrapping (1000 replicates) are indicated at the major branching points. HEs specific for 9-*O*-acetylated Sia as determined on the basis of their lectin ligand specificity and/or sialate-*O*-acetylesterase substrate preference are high-lighted in green, the one specific for 4-*O*-acetylated Sia in red. HCoV, human coronavirus; PHEV, porcine hemagglutinating encephalomyelitis virus, EqCoV, equine coronavirus.
(TIF)

Figure S2 **Electron density of the receptor.** (A) Difference electron density map calculated from the final model from which the ligand had been omitted. The contour level is 3.0 σ. (B) 2Fo-Fc map of the final model contoured at the 1.0 σ level.
(TIF)

Figure S3 **A large shift in ligand specificity through modest changes in receptor-binding site architecture.** (A) Stick representation of αNeu4,5,9Ac_3_2Me in gray with (*left*) the 5-*N*-Ac- and 9-*O*-Ac- or (*right*) the 4-*O*-Ac- and 5-*N*-Ac-groups colored in red (oxygen), blue (nitrogen) and cyan (carbon). Arrows indicate the distances between Ac methyl groups and asterisks the position of the O2 atom through which Sia would be linked to the penultimate residue of the glycan chain. The patch that accommodates the Sia-5-*N*-Ac group in BCoV-Mebus HE, and the newly formed pocket that accommodates the Sia-4-*O*-Ac group in MHV-S HE are colored in cyan. The pockets that harbor the Sia-9-*O*-Ac group in BCoV-Mebus HE and now accommodates the Sia-5-*N*-Ac group in MHV-S HE are colored in green. (B) Surface representation of the MHV-S HE receptor binding site with Neu5,9Ac_2_2Me modeled *in silico* in a topology corresponding to that in BCoV HE. The model predicts that 9-*O*-Ac-Sia will not be accepted as ligand because of (i) the spatial arrangement of the two hydrophobic pockets at too close a distance of each other and (ii) a clash of the Sia carboxylate with residues of the extended R3 loop.
(TIF)

Text S1 **Supplementary introduction; Receptor switching from 9-** ***O*** **- to 4-** ***O*** **-Ac-Sias or the other way around?**
(DOC)

Video S1 **A large shift in ligand specificity through modest changes in receptor-binding site architecture.** Only a few changes in the receptor binding site architecture of the MHV-S HE protein resulted in the specific binding of 4-*O*-acetylated sialic acid and the exclusion of 9-*O*-acetylated sialic acid.
(WMV)
