# Correction: The Murine Coronavirus Hemagglutinin-esterase Receptor-binding Site: A Major Shift in Ligand Specificity through Modest Changes in Architecture

**DOI:** 10.1371/annotation/a1e2a2e4-df03-40db-b10b-fd0cfcf78d3c

**Published:** 2012-02-22

**Authors:** Martijn A. Langereis, Qinghong Zeng, Balthasar A. Heesters, Eric G. Huizinga, Raoul J. de Groot

An initial is missing in the third author's name. The correct name is: Balthasar A Heesters. The correct citation is: Langereis MA, Zeng Q, Heesters BA, Huizinga EG, de Groot RJ , et al. (2012) The Murine Coronavirus Hemagglutinin-esterase Receptor-binding Site: A Major Shift in Ligand Specificity through Modest Changes in Architecture. PLoS Pathog 8(1): e1002492. doi:10.1371/journal.ppat.1002492

