# Supplementary figures and images for: The Murine Coronavirus Hemagglutinin-esterase Receptor-binding Site: A Major Shift in Ligand Specificity through Modest Changes in Architecture
Source: PLoS Pathog. 2012 Jan 26;8(1):e1002492. doi: 10.1371/journal.ppat.1002492 (PMC3266934; doi:10.1371/journal.ppat.1002492)

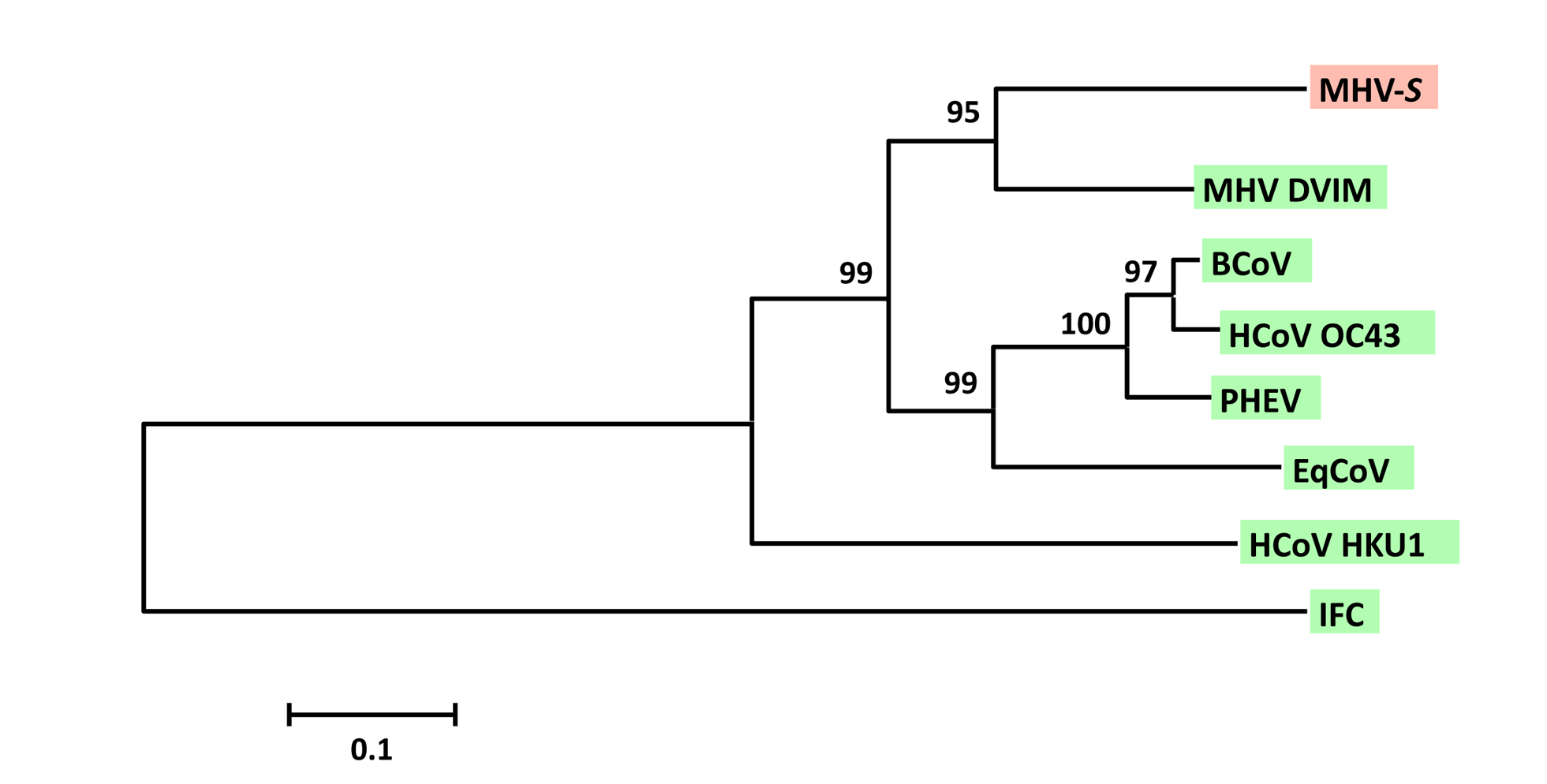

Supplement: Figure S1 — Rooted Neighbor-Joining tree depicting the evolutionary relationships among coronavirus HE proteins with Influenza C virus (IFC) HEF as outgroup. Confidence values calculated by bootstrapping (1000 replicates) are indicated at the major branching points. HEs specific for 9-O-acetylated Sia as determined on the basis of their lectin ligand specificity and/or sialate-O-acetylesterase substrate preference are high-lighted in green, the one specific for 4-O-acetylated Sia in red. HCoV, human coronavirus; PHEV, porcine hemagglutinating encephalomyelitis virus, EqCoV, equine coronavirus. (TIF) [file ppat.1002492.s001.tif]

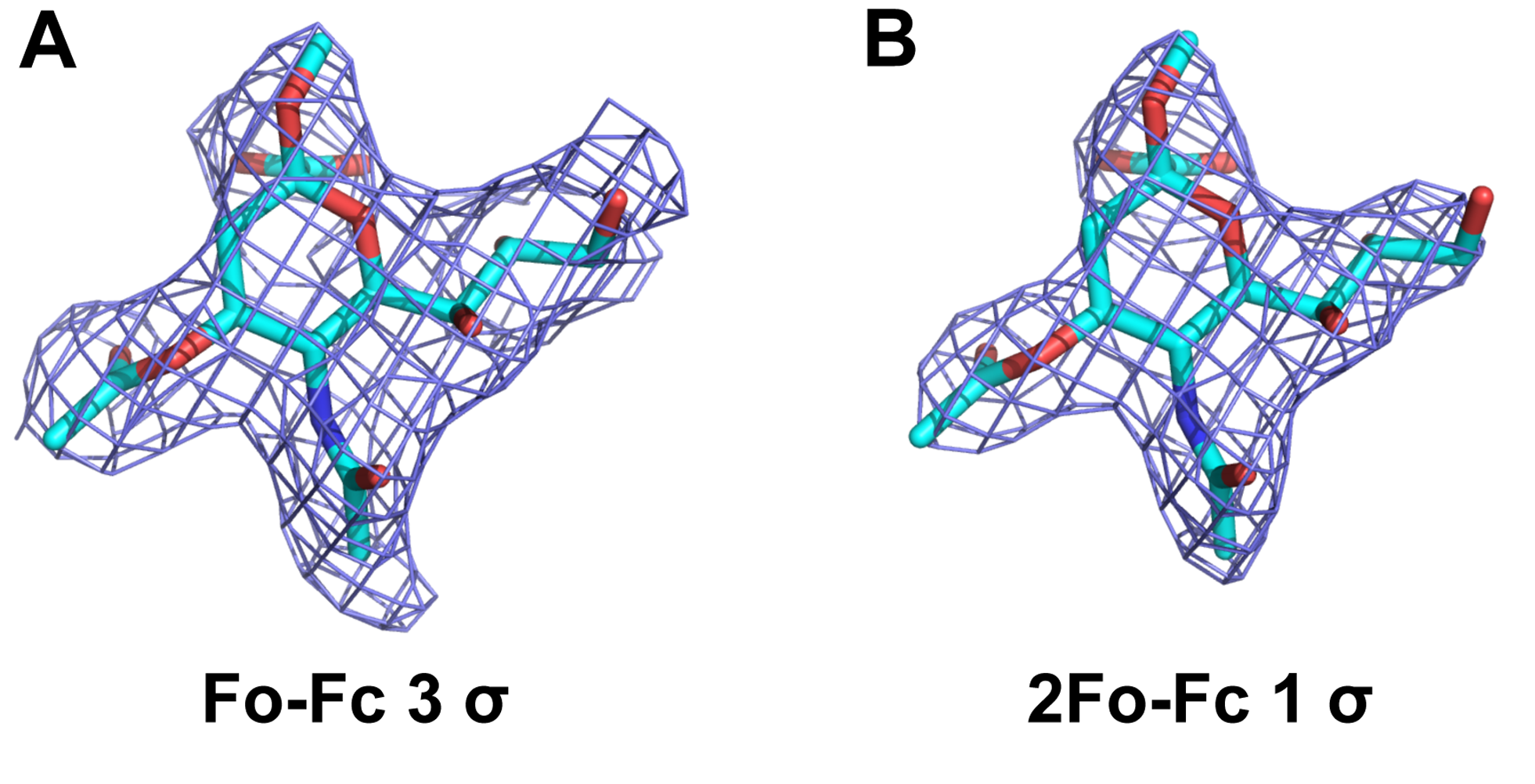

Supplement: Figure S2 — Electron density of the receptor. (A) Difference electron density map calculated from the final model from which the ligand had been omitted. The contour level is 3.0 σ. (B) 2Fo-Fc map of the final model contoured at the 1.0 σ level. (TIF) [file ppat.1002492.s002.tif]

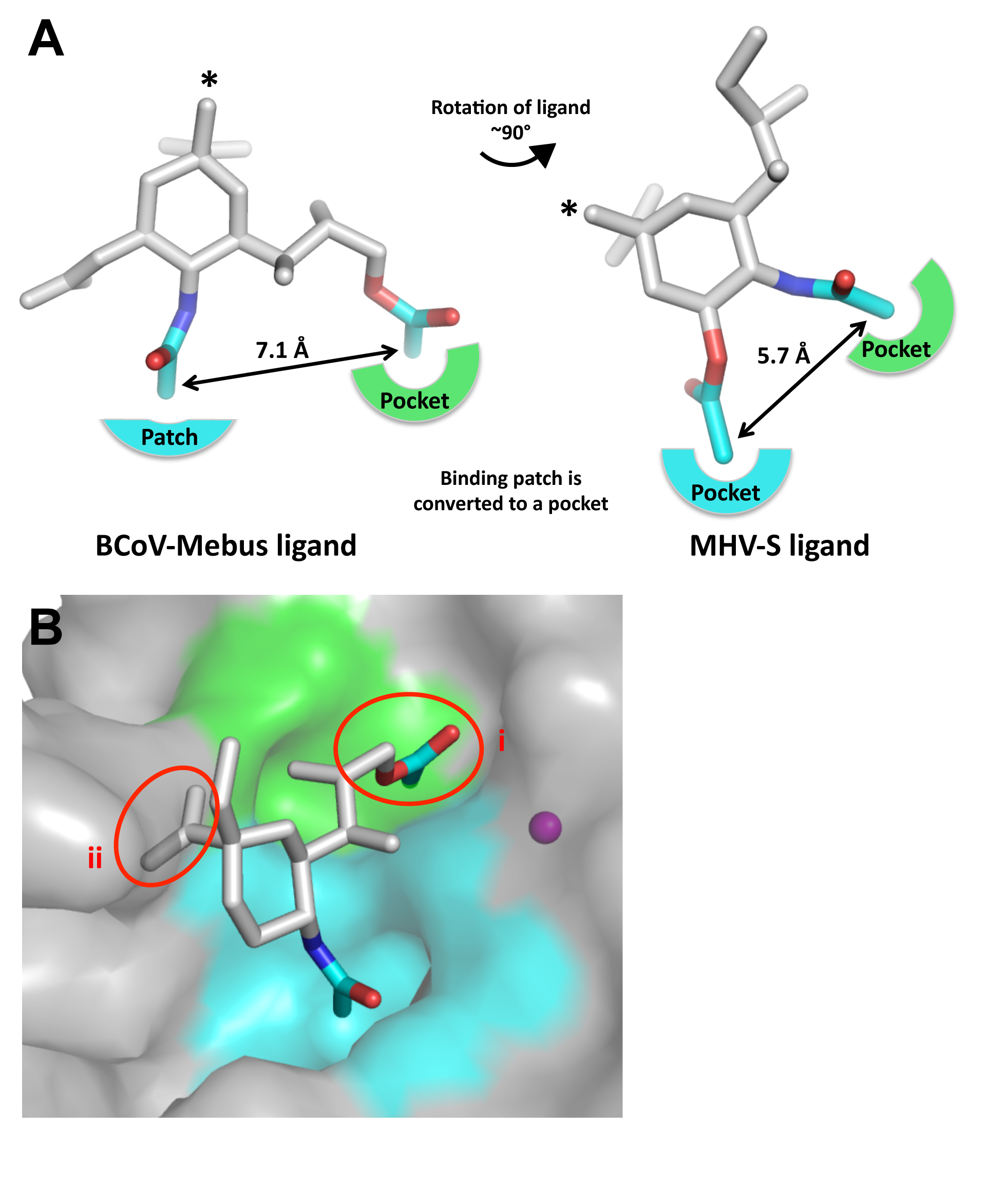

Supplement: Figure S3 — A large shift in ligand specificity through modest changes in receptor-binding site architecture. (A) Stick representation of αNeu4,5,9Ac32Me in gray with (left) the 5-N-Ac- and 9-O-Ac- or (right) the 4-O-Ac- and 5-N-Ac-groups colored in red (oxygen), blue (nitrogen) and cyan (carbon). Arrows indicate the distances between Ac methyl groups and asterisks the position of the O2 atom through which Sia would be linked to the penultimate residue of the glycan chain. The patch that accommodates the Sia-5-N-Ac group in BCoV-Mebus HE, and the newly formed pocket that accommodates the Sia-4-O-Ac group in MHV-S HE are colored in cyan. The pockets that harbor the Sia-9-O-Ac group in BCoV-Mebus HE and now accommodates the Sia-5-N-Ac group in MHV-S HE are colored in green. (B) Surface representation of the MHV-S HE receptor binding site with Neu5,9Ac22Me modeled in silico in a topology corresponding to that in BCoV HE. The model predicts that 9-O-Ac-Sia will not be accepted as ligand because of (i) the spatial arrangement of the two hydrophobic pockets at too close a distance of each other and (ii) a clash of the Sia carboxylate with residues of the extended R3 loop. (TIF) [file ppat.1002492.s003.tif]
