# Supplementary material for: The Murine Coronavirus Hemagglutinin-esterase Receptor-binding Site: A Major Shift in Ligand Specificity through Modest Changes in Architecture
Source: PLoS Pathog. 2012 Jan 26;8(1):e1002492. doi: 10.1371/journal.ppat.1002492 (PMC3266934; doi:10.1371/journal.ppat.1002492)
Supplement: Text S1 — Supplementary introduction; Receptor switching from 9- O - to 4- O -Ac-Sias or the other way around? (DOC) [file ppat.1002492.s004.doc]

**Supplementary introduction**

**Receptor switching from 9-*O*- to 4-*O*-Ac-Sias or the other way around?**

Sia specificity (lectin ligand specificity and/or sialate-*O*-acetylesterase substrate preference) has been determined for representatives of all currently known HE branches of influenza C virus, toroviruses and coronaviruses [1-3]. The HEs in all but one of these branches (the one comprising the HEs of a subgroup of murine coronaviruses) display a distinct preference for 9-*O*-Ac-Sias. These observations in combination with phylogenetic analyses of HE sequences (entailing the construction of rooted evolutionary trees; Figure S1), leave little room for scenarios other than the one that we adhere to in the manuscript, namely that the most recent common ancestor of the corona- and torovirus HEs must have been 9-*O*-Ac-Sia-specific (for a review see [6]). The alternative option that the ancestral HE displayed 4-*O*-Ac-Sia specificity would imply that the conversion from 4-to-9-*O*-Ac-Sia receptor usage occurred independently, time-and-again in toroviruses, and in influenza C viruses (for both lineages at least at single occasions) and in betacoronaviruses (at least three times). The original specificity for 4-*O*-Ac-Sias would have been exclusively maintained not in a deeply rooted branch, but rather in what appears to be one of the very most recent split-offs in the betacoronavirus HEs. Although we do acknowledge that evolution is not always linear, we consider the latter scenario highly improbable and have therefore interpreted and discussed our observations from the viewpoint that the ancestral coronavirus HE did use 9-*O*-Ac-Sias, and that a switch to 4-*O*-Ac-Sias occurred uniquely and relatively recently in the common ancestor of a single cluster of murine coronaviruses.

**References:**

1. de Groot RJ (2006) Structure, function and evolution of the hemagglutinin-esterase proteins of corona- and toroviruses. Glycoconj J 23: 59-72.

2. Smits SL, Gerwig GJ, van Vliet AL, Lissenberg A, Briza P, et al. (2005) Nidovirus sialate-O-acetylesterases: evolution and substrate specificity of coronaviral and toroviral receptor-destroying enzymes. J Biol Chem 280: 6933-6941.

3. Langereis MA (2011) Viral hemagglutinin-esterases; mediators of dynamic virion-glycan interactions. Utrecht: University of Utrecht. 174 p.
